# Functional role of cannabinoid receptors in urinary bladder

**DOI:** 10.4103/0970-1591.60440

**Published:** 2010

**Authors:** Pradeep Tyagi, Vikas Tyagi, Naoki Yoshimura, Michael Chancellor

**Affiliations:** Departments of Urology, William Beaumont Hospital, MI 48073; 1 University of Pittsburgh, PA 15213, USA

**Keywords:** Bladder, cannabinoids, irritation, protein-coupled receptor, receptor expression

## Abstract

Cannabinoids, the active components of Cannabis sativa (maijuana), and their derivatives produce a wide spectrum of central and peripheral effects, some of which may have clinical applications. The discovery of specific cannabinoid receptors and a family of endogenous ligands of those receptors has attracted much attention to the general cannabinoid pharmacology. In recent years, studies on the functional role of cannabinoid receptors in bladder have been motivated by the therapeutic effects of cannabinoids on voiding dysfunction in multiple sclerosis patients. In this review, we shall summarize the literature on the expression of cannabinoid receptors in urinary bladder and the peripheral influence of locally and systemically administered cannabinoids in the bladder. The ongoing search for cannabinoid-based therapeutic strategies devoid of psychotropic effects can be complemented with local delivery into bladder by the intravesical route. A greater understanding of the role of the peripheral CB_1_ and CB_2_ receptor system in lower urinary tract is necessary to allow the development of new treatment for pelvic disorders.

## PHARMACOLOGY OF PHYTOCANNABINOIDS GUIDING RECEPTOR DISCOVERY

**Figure 1:**
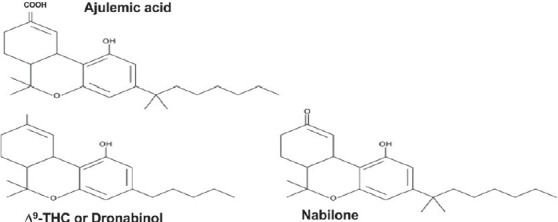
Chemical Structures of classical synthetic cannabinoids, with a basic tricyclic dibenzopyran structure, which is also shared by the psychoactive principle (-) Δ^9^-THC from cannabis. Synthetic analog of (-) Δ^9^-THC is the drug known as dronabinol. Two other drugs nabilone and ajulemic acid are synthetic derivatives of a phase I metabolite of (-) Δ^9^-THC. They differ from (-) Δ^9^-THC in terms of the extra methyl groups and a ketonic or carboxyl group in place of methyl group at the ninth carbon position

For many centuries, phytocannabinoids obtained from cannabis plant (mar?uana) have been consumed for their analgesic, anxiolytic, antiemetic and antispasmodic properties especially in the oriental culture.[1] However, therapeutic utility of cannabis plant or its products in the evidence based medicine continues to remain a lightning rod for controversy on social, legal and medical fronts. Pharmacological and chemical investigation on cannabis plant found more than 50 compounds, of which the main psychoactive principal was identified as Δ^9^-tetrahydrocannabinol, ( Δ^9^-THC) [Figure 1] apart from two other bioactive cannabinoids, cannabidiol (CBD) and cannabinol (CBN).[2] Further studies dealing with the search of biological targets for the Δ^9^-THC led to the cloning and identification of two CB_1_ and CB_2_ receptors belonging to the heptahelical G protein-coupled receptor (GPCR) superfamily.[3]

The receptor CB_1_ is the most abundant of all receptor types in the brain and other CNS regions involved with pain transmission and modulation, specifically in the spinal dorsal horn and periaqueductal gray.[4,5] CB_1_ receptors are also located peripherally in both neuron and non-neuronal tissue, while CB_2_ receptors are mainly found in immune cells and brain glial cells.[4,6] These receptors have been found to have many physiological and patho-physiological functions, including mood alteration, control of feeding and appetite, motor and co-ordination activities, analgesia, immune modulation and gut motility.[1]

Given the ubiquitous expression of CB_1_ and CB_2_ receptors, cannabinoids have been shown to produce wide spectrum of effects including induction of proliferation, growth arrest, or apoptosis in a number of cells, including neurons, lymphocytes, and various neural and non neural cells.[7] Alterations in the reproductive system produced by cannabis motivated the studies leading up to the discovery [8] CB_1_ receptors have been detected in the testis, prostate and vas deferens.[9–11] In addition, expression of functional CB_1_ receptors on sperm and presence of the archetypal endocannabinoid anandamide in reproductive secretions have also been detected [12] It can be said therefore that discovery of cannabinoid receptors in bladder lagged behind the discovery of these metabotropic receptors in other organs lining the genitourinary tract.

### CB_1_ and CB_2_ receptors and signaling

**Figure 2:**
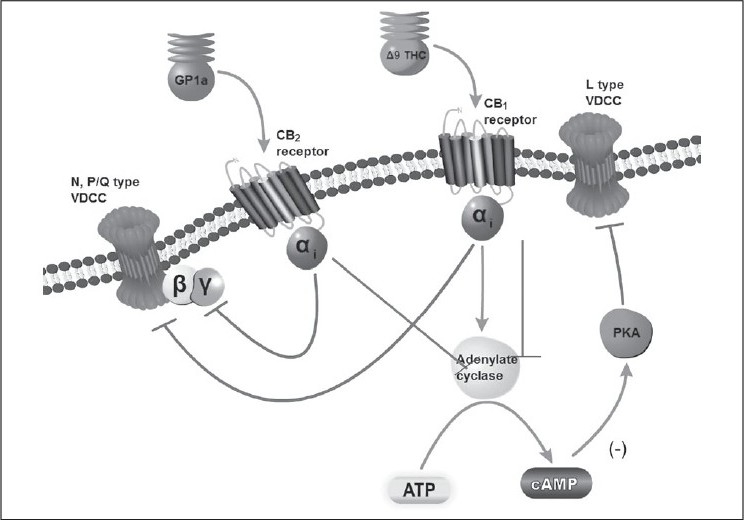
The metabotropic CB_1_ receptor exhibit 48% amino acid sequence identity with CB_2_ receptors and both of them are negatively coupled to adenylyl cyclase to inhibit cyclic AMP that indirectly inhibit L-type Ca ^2 + ^ channel. CB_1_ receptors couple via pertussis toxin -sensitive Gi/o proteins to inhibit N^−^, and P/Qtype Ca^2 + ^ channels and K + channels. The inhibitory effects of cannabinoids on Ca^2 + ^ channels in nerve terminals are similar to other endogenous anti-nociceptive agents such as opioids.[14] GP1a (N-(Piperidin-1-yl)-1-(2,4-dichlorophenyl)-1,4- dihydro-6-methylindeno[1,2-c]pyrazole-3-carboxamide) is a highly selective CB_2_ agonist with Ki values of 0.037 and 363 nM for CB_2_ and CB_1_, respectively. The Δ^9^-THC mimics the action of endocannabinoids and acts non-selectively on both CB_2_ and CB_1_ receptors

Cannabinoids elicit their well known diverse effects by activating numerous signaling pathways. CB_1_ and CB_2_ receptors exhibit 48% amino acid sequence identity and both of them are negatively coupled to adenylase cyclase to inhibit cyclic AMP and mitogen-activated protein kinase [Figure 2].[13] In addition, CB_1_ receptors couple via pertussis toxin -sensitive G_ig/o_ proteins to inhibit L-, N-, and P/Q-type calcium channels and activate potassium channels.[14] The endogenous ligands for these receptors are called as endocannabinoids (ECB), which are namely anandamide, 2-arachidonoylglycerol, virodhamine, and noladin ether (2-arachidonylglyceryl ether).

These ECBs structurally resemble eicosanoids as they are derived from arachidonic acid; a polyunsaturated fatty acid that serves as precursor for a plethora of other bioactive metabolites such as prostaglandins, thromboxanes, leukotrienes etc. In animal studies, the pharmacological action of Δ^9^-THC was mimicked by endocannabinoids.[15] Most of the ECBs derived from arachidonic acid act in a receptor-dependent manner and have the ability to act as retrograde inhibitors of synaptic neurotransmission in GABAergic and glutamatergic synapses, as well as modulators of post-synaptic transmission, involving norepinephrine and dopamine.[14] These ECBs are transported into cells by a specifi c uptake system and degraded by two well-characterized enzymes, fatty acid amide hydrolase (FAAH) and monoacylglycerol lipase.[16] The ECBs are synthesized on demand and have short-lived effects due to effective metabolic pathways.[17]

ECBs, like anandamide, are different from classical neurotransmitters in the sense that they are not stored in and released from nerve vesicles, but rather released on demand from the nerve cell membrane during inflammation.[18] The anandamide released from post-synaptic cells could mediate its inhibitory effect by acting on presynaptic CB_1_ receptor. The inhibition of transmitter release from nociceptive afferents by anandamide may indicate a mechanism for modulating the spinal nociceptive pathways.[19] The growing knowledge of the broad physiological roles of the ECBs including biosynthesis and catabolism is providing insight into potentially novel therapeutic targets.[20]

### Expression of cannabinoid receptors in bladder

The results of a recently completed large randomized, controlled, multicenter clinical trial known as the cannabinoids in multiple sclerosis study (CAMS) sparked the interest in studying the expression of cannabinoid receptors in resident bladder tissue of urothelium and detrusor.[21] The multi-center CAMS study randomized 630 patients to receive either oral administration of cannabis extract, Δ^9^-THC or a matching placebo. Patients completed incontinence diaries throughout the study. Significant reduction in urge incontinence episodes and improvement in bladder control from baseline were noted at the end of the study with the use of cannabis extract (38%) or Δ^9^-THC (33% reduction).[21] The small increase in efficacy of cannabis extract over pure Δ^9^-THC seems to suggest that ingredients other than Δ^9^-THC in cannabis extract such as CBD and CBN may antagonize some of the undesirable effects of Δ^9^-THC and contribute positively to bladder symptoms.[22] The placebo arm of the trial only showed 18% decrease in incontinent episodes relative to baseline to further suggest a distinct clinical effect of cannabinoids on bladder symptoms.

### Cannabinoid receptors in rodent bladder

In earlier studies, the presence of CB_1_ receptors has been indirectly demonstrated in the rodent bladder using specific [23] Results from an isolated bladder strip study suggested that these receptors are located in the prejunctional neuron. Further, systemic administration of CB agonist and antagonists in spinal cord injured rats with detrusor overactivity demonstrated the role of a tonically active ECB system in pathological voiding.[24]

Previously, the expression of muscarinic, neurokinin and beta 3 adrenoceptors in bladder have been successfully demonstrated using molecular and pharmacological techniques.[25] Literature accounts on the expression of CB_1_ and CB_2_ receptors in different organs have relied on differentapproaches, such as autoradiography, in situ hybridization of receptor messenger RNA[26] or functional assays.[27] A recent study determined the localization of CB_1_ and CB_2_ receptors in rat bladder by immunohistochemistry and a functional assay.[28]

Hayn *et al*., positively identified the expression of CB_1_ receptors in rat bladder by the immunoreactivity of CB_1_ bladder comparable to that in cerebellum. Similarly, the presence of immunoreactivity for CB_2_ in spleen was used as positive control for the positive localization of CB_2_ receptors in bladder by the same antibody.[28] The known ability of peripheral cannabinoid receptors to modulate afferent transmission by modulating the stimulus-evoked neuropeptide release was used to design a functional assay.[29] Studies show that quantification of released calcitonin gene related peptide (CGRP) can be a suitable marker for measuring afferent neuronal activity in rat bladder.[30] The bladder of adult female rats receives approximately 16,000 axons (i.e., is the target of that many ganglion neurons) of which at least half are sensory.[31] Virtually all bladder sensory fibers that originate from dorsal root ganglia are immunoreactive for capsaicin receptor transient receptor potential vanilloid (TRPV1) and CGRP.[32] The role of TRPV1 in voiding has been well established[33] but the potential role of CB_1_ and CB_2_ receptors in micturition and pain originating from bladder is yet to be completely investigated.

**Figure 3:**
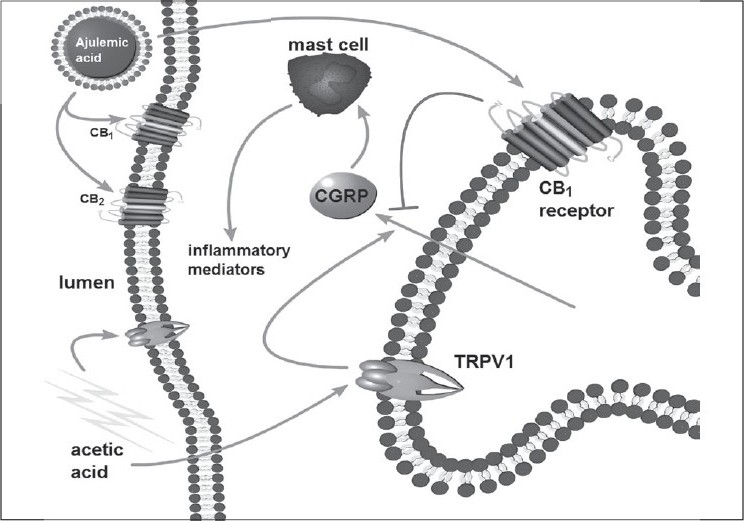
Proposed mechanism of locally administered cannabinoid agonist in irritated bladder. Bladder irritated by acetic acid, in the animal model, activates TRPV1 on urothelium and adjoining nerves to release CGRP. Irritation evoked release of CGRP is blocked by mixed CB_1_/CB_2_ agonist ajulemic acid entrapped into liposome that activates CB_1_ and CB_2_ receptors on bladder surface and nerves

The presumed expression of CB receptors on capsaicin- sensitive sensory nerves,[34] being coupled to inhibition of neurotransmitter release, was demonstrated in an isolated rat bladder model. Application of the mixed CB_1_/CB_2_ receptor agonist, ajulemic acid (AJA) inhibited the evoked release of CGRP from afferent nerve terminals in isolated rat bladder. Sensory afferent axons in the bladder are the only structures in the bladder that contain high levels of CGRP released upon nerve depolarization or chemical stimulation by capsaicin [Figure 3].[35] Pharmacological specificity of the inhibitory effect of AJA on sensory neuronal activity originating from the bladder through CB_1_ and CB_2_ receptors was demonstrated using selective receptor antagonists.

### Cannabinoid receptors in human and primate bladder

**Figure 4:**
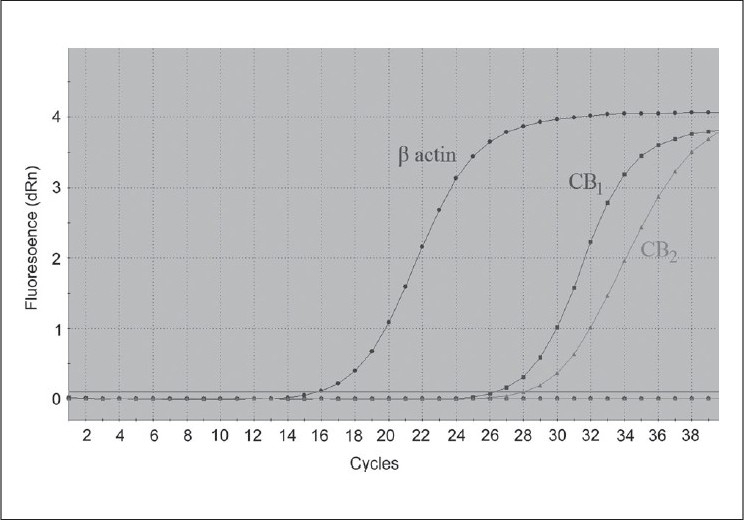
Amplification plot from a typical real time PCR experiment to detect expression of CB receptors in human urothelium relative to housekeeping gene β- actin. The urothelium specimens were obtained from organ donors. M3000P instrument measures the fl uorescence of dsDNA intercalating dye SYBR Green twice in each PCR cycle. This infl ection point is called the cycle number (Ct) at which fl uorescent signal generated passes over threshold baseline. The Ct value was determined for each specimen to measure receptor expression

Encouraged by expression in rat bladder, the expression of CB_1_/CB_2_ receptors in bladder obtained from human cadavers was recently investigated using different techniques.[36] Expression of functional CB_1_ and CB_2_ receptors in human detrusor and urothelium was demonstrated using real-time quantitative polymerase chain reaction QPCR and protein expression using immunohistochemistry and Western blot. QPCR was done using customized CB_1_ and CB_2_ primers which amplified gene products from the open reading frame of single exon human CB_1_ and CB_2_ genes [Figure 4]. Expression of CB_1_ and CB_2_ receptors was demonstrated in the detrusor and urothelium, with the expression for both receptors approximately two fold higher in the urothelium than in the detrusor (*P* < 0.05). The mRNA expression of the CB_1_ receptor was significantly higher than that of the CB_2_ receptor in both tissue types (*P* < 0.05). Immunofluorescence results show that expression of CB_1_ and CB_2_ is specific to bladder and not contributed by infiltrating cells.

Expression of CB_1_ and CB_2_ receptors detected at the mRNA level by QPCR was confirmed at the protein level by immunoreactivity and Western blot analysis. Activation of CB_1_ and CB_2_ receptors attenuated the electrically evoked contraction of detrusor strips. These inhibitory effects of cannabinoid receptor agonists were suggested to be attributable to prejunctional CB_1_ receptors that decrease contractile transmitter release.[36] It is well accepted that endogenous cannabinoids and CB_1_ receptors are involved in the regulation of smooth muscle contractility, through a mechanism mainly related to reduction of acetylcholine release from cholinergic nerve endings.[37] The modulatory action of the cannabinoid agents on the non-adrenergic non-cholinergic neurotransmission operating in the bladder is yet to be studied.

Using similar techniques of Western blot and immuno histochemistry, similar results on expression of CB_1_/CB_2_ receptors in human bladder were also reported by Gratzke et al., 2009.[34] Apart from humans, this group also investigated the distribution of CB_1_ and CB_2_ receptors in the rat and monkey species. Higher expression of CB_2_ receptor, but not CB_1_, was noted in the urothelium relative to detrusor. Expression of CB_2_ receptors in urothelium was localized to the sensory and cholinergic nerves in the bladder obtained from humans as well as other species of rats and rhesus monkeys. Co-localization of CB_2_ receptor antibody stain with the stain for CGRP, TRPV1, and vesicular acetylcholine transporter (VAChT) protein specific for cholinergic nerves further confirmed the expression of CB_2_ receptor by bladder afferents. Nerve fibers containing CB_2_ and VAChT were also located in the detrusor. The co-expression of VAChT and CB receptor, and effects by CP55940, on nerve mediated contractions suggest a CB_2_ receptor mediated modulatory effect on cholinergic nerve activity in bladder.[34]

The localization of CB_2_ receptor with nerves argues for a role of CB_2_ in bladder afferent signals which can be best demonstrated by cystometric studies. These *in vivo* effects of CP55940 (CB_1_/CB_2_ receptor agonist) on urodynamic parameters. MI and TP may be considered parameters that indirectly represent sensory functions during cystometry. Lack of a direct effect of CB_1_/CB_2_ agonist CP55,940 and the nonselective agonist anandamide on carbachol induced contractions indicated the absence of direct CB mediated functions of isolated detrusor smooth muscle.[34]

Recent studies demonstrated the modulatory effects of CB_1_/CB_2_ agonists on nerve induced contractions in detrusor preparations obtained from most mammalian species.[23,28,34,36] In contrast, according to a previous report CB_1_/CB_2_ agonists fail to show any effect on the electrically evoked contractions of bladder preparations isolated from dogs, pigs, cynomolgus monkeys and humans.[27] However, the same report was able to reproduce the inhibition of neuronally evoked contractions in isolated rat and mouse bladders by CB_1_/CB_2_ agonists. Definitive characterization of CB receptor in tissues depends on the availability of selective agonists and antagonists and difference in the selectivity of agents used in different studies may explain the different results. In addition, there may be differences in the age and associated pathology of organ donors who donated the bladder for the muscle strip studies. Previously published studies in the spinal cord injured rat revealed that peripheral CB receptors are involved in detrusor over activity.[24] The inhibitory effect of CB agonists on detrusor pressure observed by Blyweert *et al.,* further corroborates the inhibitory effects of CB agonists on isolated human detrusor strips in organ bath.

### Unique pharmacology of anandamide in bladder

One of the interesting aspects of study reported by Gratzke et al., 2009.[34] is the difference in the pharmacology of a synthetic CB_1_/CB_2_ receptor agonist and ECBs such as anandamide. The difference was best illustrated by anandamide led 26% increase in the TP and 19% decrease of MI in cystometric studies relative to increase of 124% (TP) and 46% (MI) by CP55940 (*P* < 0.05 and < 0.01, respectively). Furthermore, nerve mediated contractions were enhanced by anandamide and decreased by CP55940 (*P* < 0.05).[34]

The unique pharmacology of anandamide have been explained in the past by its ability to elicit effects by acting on both via G protein-coupled CB_1_ receptors and transient receptor potential (TRP) channels (chiefly TRPV1 receptors). The pre-synaptic inhibitory effect of anandamide is evident at low concentration and at higher concentrations the activation of TRPV1 counters the effect on CB_1_/CB_2_ receptors.[19] The activation of TRPV1 channels can lead to desensitization and loss of pre-synaptic inhibition observed in various studies.[28]

Anandamide has also been noted to aggravate cyclo phosphamide induced cystitis in rodents through its activation of TRPV1 ion channels and thereby causing detrusor overactivity and hyperalgesia.[38] In contrast, anandamide have been also shown to mediate attenuation of detrusor overactivity induced by nerve growth factor instillation in bladder.[39] The attenuation of detrusor overactivity by anandamide unmasked the role of CB_2_ receptors expressed in bladder in controlling the pain initiated locally in bladder. [40] Concentration and time of exposure may be critical in determining which of these opposite effects of anandamide ultimately prevails.

Most TRPV1 receptor-expressing cells are also known to co-express the CB_1_ receptors as well. [41] The close proximity of CB_1_ and TRPV1 may facilitate the dual, concentration- dependent effect of anandamide observed in different studies.[38] Dual dose dependent effect of cannabinoids have also been previously noted in relation to immune system where low doses of cannabinoids may enhance cell proliferation and high doses of cannabinoids may induce growth arrest or apoptosis.[7] The ability of same ligands to activate both metabotropic CB_1_/CB_2_ receptors and ionotropic TRPV1 receptors suggest possible interactions between the two signaling systems.[38] Stimulation of CB_1_ and desensitization of TRPV1 could be a strategy to protect against inflammation in bladder.

To further confound the pharmacology of anandamide in bladder, the responses to anandamide as reported by Gratzke et al., (2009) were attenuated but not abolished after desensitization by capsaicin. Further, anandamide response were partially attenuated by an prostaglandin receptor EP1 antagonist and almost abolished by indomethacin, a cyclooxygenase inhibitor.[34] Neither the CB_1_ antagonist AM251 nor the CB_2_ antagonist AM630 had any effect on the response to anandamide, to suggest possible role of EP1 receptor.

### Cannabinoid receptors fueling drug discovery

The distribution of these CB_1_/CB_2_ receptors at key sites involved in nociceptive processing is instrumental in the analgesic effects of phytocannabinoids (plant source) or synthetic cannabinoids developed in last 30 years.[13] Synthetic cannabinoids are chemicals having action similar to cannabis on their cognate receptors. Studies have shown that synthetic and semi-synthetic cannabinoids that lack psychotropic effects are effective against severe pain states refractory to even opioids.[42] The activation of nociceptive sensory neurons leads to nociception. However, CB_1_/CB_2_ agonists are capable of altering nociceptor activity without producing nociceptive behavior.[13,43] CB_1_ /CB_2_ agonists have been able to suppress the nociceptive transmission and inhibit pain-related behavior in animal models of acute and persistent nociception by their activity at spinal, supraspinal and peripheral sites.[44]

The new drugs based on pharmacology of cannabinoids can be classified into two categories: Direct and indirect agonists. Direct agonists selectively activate either CB_1_ or CB_2_ receptor. CB_2_ receptor agonists are not associated with the adverse side-effects of CB_1_-selective agonists and therefore may provide an alternative analgesic target.[45] Indirect agonists work on the principle that metabolic degradation is the rate-limiting step in the therapeutic effects of ECB and the efficacy can be magnified by blocking the ECB metabolism either through cellular reuptake or enzymatic hydrolysis. Such compounds can theoretically act selectively on tissues with ongoing synthesis and degradation of ECB, thus producing fewer unwanted effects than direct agonists. By acting through up-regulation of ECB, another advantage of indirect agonists is that they may produce beneficial actions through actions on other receptors as well such as CB_1_, CB_2_ or TRPV1 receptors.

The drug discovery of synthetic cannabinoids is also fuelled by the notorious toxicity of cannabis or phytocannabinoids. Some synthetic cannabinoids with limited side effects and abuse liability have already been approved for clinical use in Canada. Nabilone and dronabinol are classical synthetic cannabinoids, with chemical structure based on Δ^9^-THC, approved for treating severe nausea and vomiting associated with cancer chemotherapy[15] [Figure 1]. A plethora of preclinical reports on analgesic effect of cannabinoids[44] has motivated the off-label use of nabilone and dronabinol in chronic pain management in a few clinical trials, case reports or case series.[46,47] To avoid the possible risk of abuse of these drugs by patients, experts in the field have put guidelines for clinical use of these agents.[48]

These peripherally acting agents could evoke profound pain relief in animal models as well as in a few small clinical studies, but the underlying mechanism and signaling pathways mediating these effects are yet to be completely understood.[44] A major challenge facing the biomedical research community is the identifi cation of compounds that are safe and effective in treating pain, particularly chronic pain such as painful bladder syndrome (PBS) or interstitial cystitis (IC). Various methods, medicines, and devices are available to IC/PBS patients to reduce their pain and symptoms but many of these conventional therapies have significant limitations.[30] Based on the known effects of cannabinoids, in preclinical and clinical studies, it can expected that peripherally acting cannabinoid receptor agonists can modulate bladder sensory pathways by acting on nociceptive pathways originating from bladder.

### Route of administration affects bioavailability and toxicity of cannabinoids

As described in earlier sections here, experimental factors such as drug concentration, timing of drug delivery and location of drug administration can influence the therapeutic and adverse response of cannabinoids. The acute adverse effects reported with the consumption of cannabis by smoking or by oral route includes increased food intake, tachycardia, orthostatic hypotension, pulmonary irritation, impaired motor coordination, cognitive impairment, anxiety, paranoia, and psychosis.[49] In addition, the bioavailability of CBs from the oral route is uncertain as illustrated by unpredictable pharmacokinetics of (Δ^9^- THC) after oral administration.[50] This has generated a lot of interest in alternative routes for delivering cannabinoids. It was recently shown that topical application of 30 µg of Δ^9^-THC reduced allergic inflammation in mouse ear model of allergic dermatitis.[51] The activation of cutaneous CBs lead to attenuation of nociceptor excitation, pain and itch perception,[52] and decreased the release of neuropeptides, particularly CGRP, from terminal afferents

It is definitely more logical to develop local CB delivery with predictable bioavailability that rules out central side effects. Route of inhalation is also a distinct possibility for the therapeutic delivery of cannabinoids, as shown by the recent approval of an oromucosal (sublingual) spray standardized for the Δ^9^-THC and CBD (1:1 ratio) in Canada, as adjunctive treatment for neuropathic pain of multiple sclerosis patients. (53) The rationale for the combination of CBD and Δ^9^-THC is that CBD can antagonize some undesirable effects of THC including intoxication, sedation and tachycardia, while contributing analgesic, anti-emetic properties.[22] This is because Δ^9^-THC can activate cannabinoid CB_1_ and CB_2_ receptors but CBD possesses no, or very weak affinity for these receptors. The spray is administered with a device equipped with an electronic tool to facilitate accurate dosing.

In an open-label pilot study on advanced MS patients, daily inhalation from the oromucosal spray (Sativex^®^) for 16 weeks improved the refractory lower urinary tract symptoms of these patients.[54] The spray also reduced neuropathic pain, spasticity, muscle spasms and sleep disturbances. Most common adverse events reported were dizziness, sleepiness, fatigue, feeling of intoxication and a bad taste.[55]

### Local administration of cannabinoids inside bladder

Studies done on other tissues such as ear and paw have already shown that locally administered CB agonists act on peripheral receptors and attenuate the pain behavior induced by localized tissue damage or irritation.[40,51,56] Local administration of anandamide via intraplantar injection suppressed neuropathic pain in rats.[57] These observations lend support to the concept that CB receptors in the periphery participate in the intrinsic control of pain initiation and locally generated endocannabinoid such as anandamide and may mediate this effect.[40] Recent studies indicate a possible antinociceptive synergy from cannabinoid action on peripheral receptors with that on spinal sites.[56]

Intravesical drug delivery offers an attractive opportunity to focus the potency of potentially toxic drugs only on site of action as demonstrated by clinical management of PBS/ IC with intravesical DMSO.[58] In recent years, considerable effort has been expended into developing formulation suitable for intravesical administration of antimuscarinics, capsaicin, resiniferatoxin and local anesthetics.[58,59] The success of delivering antimuscarinics, capsaicin and botulinum toxin into the bladder,[60,61] encouraged intravesical delivery of cannabinoids using liposomes.[62–64] Phytocannabinoids and synthetic cannabinoids are generally not soluble in water and formulation of these agents into liposomes can overcome the aqueous solubility of these agents.[62–64]

Guided by the results of CGRP experiments in isolated bladder, selective CB_1_/CB_2_ agonist AJA was loaded inside the liposomes and instilled into bladder to unmask the role of peripheral cannabinoid receptors in bladder. AJA is a synthetic derivative of nabilone that is currently approved in Canada for chemotherapy induced emesis. AJA has been efficacious in animal models of chronic pain by activation of the CB_1_ receptor with a superior therapeutic index compared to other CB compounds.[65] AJA binds to human CB_1_/CB_2_ receptors *in vitro*, with high affinity at human CB_1_ (Ki 6nM) as well as h CB_2_ (Ki 56 nM) receptors.[65] In a previous study, by our group, on systemic administration of AJA, the role of CB_1_/CB_2_ receptors in micturition was more evident in the irritated condition of bladder than in normal condition.[66]

**Figure 5:**
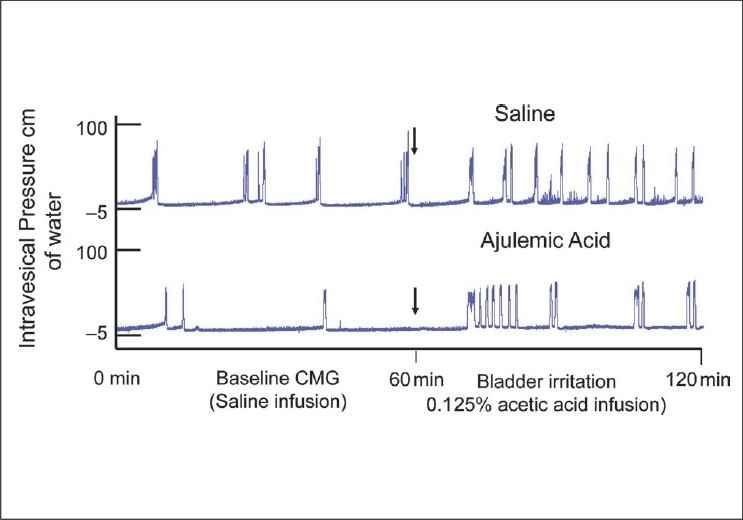
Cystometric effect of mixed CB_1_/CB_2_ receptor agonist, ajulemic acid on bladder irritation induced by infusion of acetic acid (0.125%). Rats were pretreated with either saline or ajulemic acid entrapped in liposomes. Baseline cystometry (CMG) was done under saline infusion prior to bladder irritation induced by acetic acid. Activation of local cannabinoid receptors in the bladder blunted the decrease in micturition interval MI induced by acetic acid. Decrease in MI is evident from reduced time interval between the peaks of cystometrogram of saline treated rat relative to rat treated with ajulemic acid. The urodynamic parameter, MI, indirectly represents sensory functions and reduced MI is an integrated response of irritated bladder

Female rats were pretreated with AJA (0.5 ml of liposomal for 30 min) prior to irritation with acetic acid [Figure 5]. First, baseline cystometric parameters were derived through transurethral open cystometry (CMG) under urethane anesthesia (dose1.0 g/kg body weight), with saline infusion at the rate of 0.04 ml/min. Bladder irritation was induced by infusing acetic acid (0.125% v/v) in saline into the bladder.[64] As shown in the CMG tracing of Figure 5, infusion of acetic acid in bladder reduces the micturition interval MI of rats instilled with saline previously, because acetic acid irritates the afferents in the bladder to induce hyperexcitability.

As revealed by cystometric parameters, local activation of cannabinoid system in bladder by intravesical administration can blunt the pain initiated in the bladder by acid infusion. The therapeutic effect of instilled drugs was assessed by the per cent reduction in MI after infusion of acetic acid. Cystometric data supported the hypothesis that instilled CB_1_/CB_2_ agonist can buffer the pain signals emerging from bladder following acetic acid infusion. Local action of CB_1_ on sub agonist in the bladder may involve action at CB_1_ receptors on sub urothelial nerve plexus to reduce afferent excitability induced by acetic acid [Figure 4].

Local action of CB agonist in the bladder may involve action at receptors on peripheral nerves or an indirect modulation of immune cell responses decreasing peripheral nerve excitability [Figure 5]. Similar pharmacological studies in gut have shown that activation of CB_1_ receptor inhibits intestinal motility by reducing the acetylcholine release from enteric nerves.[67] Possibility of a similar action of CB_1_ agonists in bladder, to explain higher MI, cannot be ruled out. Locally administered cannabinoids can also inhibit NF-kB activation to suppress hyperalgesia.[68] These findings support the notion that cannabinoid system participates in buffering the pain signals emerging from the peripheral sites.

The beneficial effects of direct agonists of CB_2_ receptor instilled locally in the bladder can also be explained by action on bladder mast cells that make important contribution to the visceral hyperalgesia initiated by local irritation.[69] Expression of CB_2_ receptor have previously been documented in mast cells and local injection of CB_2_ receptor agonist JWH-133 provided analgesic effect in models of acute, inflammatory and neuropathic pain.[70] Release of inflammatory mediators from inflamed tissue is purported to be suppressed by activation of CB_2_ on non neuronal cells.[69] Alternative mechanisms that may explain these observations include attenuation of NGF-induced mast cell degranulation and neutrophil accumulation in bladder cannot be ruled out.

The property of CB_1_ agonists to evoke desensitization without nociception has unrealized potential. Studies have indicated that opioids and cannabinoids act at different systemic or peripheral sites to produce antinociception through independent mechanisms. No evidence of any cross-tolerance between the antinociceptive effects of opioids and cannabinoids was seen in animals.[44] It is well recognized that the opioids, a powerful class of analgesics which have been long utilized in clinical pain management, are not effective against neuropathic pain and are amenable to tolerance development.[71] Chronic pain may induce changes in gene expression at the site of inflammation that makes the pain more responsive to local treatment, just as local administration of opioids could only evoke dose-dependent naloxone-reversible analgesia in patients suffering from chronic pain but not from acute inflammatory pain.[72]

Studies on intravesical administration of CB agonists demonstrate that it is ideally suited to best maximize the beneficial effects of cannabinoid agents and avoid the possibility of dysphoric and psychotropic side effects. The studies demonstrating the presence of CB_1_ and CB_2_ receptor subtypes in the human bladder may open up the possibility of deriving therapeutic benefit in patients with IC/PBS by activating these receptors. Given their role in pain transmission, we surmise that CB_1_ and CB_2_ receptors are expressed at higher levels in the bladder of patients with IC/ PBS. Future studies using bladder tissue from human subjects with IC/PBS could be performed to test this hypothesis and further validate the use of CB agents for genitourinary pain. CB based drugs acting through novel therapeutic target and mechanism can be a new approach for managing pain associated with IC/PBS.

### Knockout mice of cannabinoids receptors

It can be a challenge to determine the *in vivo* mechanism of CB agonists administered systemically or intravesically[73]given the ability of CB ligands to activate other receptors, namely TRPV1, at varying concentrations. This can also explain the difficulty faced by researchers engaged in investigating the role of CB_1_ and CB_2_ receptors using pharmacological antagonists and agonists. For example, SR-141716, a CB_1_ receptor antagonist, can also show agonist property because of its other effects.[74] Therefore, the use of a genetic approach has gained favor among scientists to complement the pharmacological analysis of the cannabinoid system, and mice with targeted deletions in the cannabinoid receptor genes have been generated to study role of of the endocannabinoid system in addiction research.[75] The pharmacological specificity of CB_1_/CB_2_ agents administered systemically or intravesically can be easily determined by comparing results in knockout mice with wild type littermates. The development of transgenic CB_1_^-/-^ and CB_2_^-/-^ receptor knockout mice using homologous recombination has opened up the opportunity to study the role of the CB_1_ and CB_2_ receptor system in lower urinary tract. The concerns of global deficit of CB_1_ and CB_2_ receptors, in the survival of these mice, can be ameliorated using time-dependent and bladder specific deletion of CB_1_ and CB_2_ receptors.

### Take home message and important points

1. Expression of CB_1_ and CB_2_ receptor in lower urinary tract is relevant to effects of cannabinoids on voiding dysfunction.
2. Expression of CB_1_ and CB_2_ receptor in bladder demonstrated by molecular, immunofluorescence, detrusor strip contraction and cystometric studies.
3. Pharmacology of ECBs is complex due to their ability to act on multiple receptors.
4. Route of administration have a drastic influence on therapeutic index of of cannabinoids.

## CONCLUSIONS

The diverse effects of CB_1_ and CB_2_ receptor system in lower urinary tract may be novel targets for therapies designed to treat diseases afflicting lower urinary tract. The growth-inhibiting action of cannabinoids acting on these receptors expressed on transformed cells might be useful for the management of malignancy in bladder. Recently published pre-clinical studies have demonstrated that cannabinoids appear to act principally as prejunctional modulators of neurotransmission to affect the micturition process indirectly by affecting the nociceptive responses pathways. It is likely that CB_1_ and CB_2_ receptors located in periphery such as in bladder participate in the intrinsic control of initiation of afferent stimulus. Emerging studies show that ECBs are mediators of spinal activity-dependent pain sensitization to create a future role for pharmacological antagonists CB_1_ and CB_2_ receptors in control of neuropathic pain.

Historically, products from cannabis or its synthetic analogues have faced many obstacles in getting investment from the pharmaceutical industry and acceptance from regulatory agencies. Currently, opioids are the most effective prescription-based analgesics for the painful symptom emanating from the lower urinary tract with limited efficacy and serious toxicity, such as tolerance development, physical dependence, sedation, respiratory depression and gastrointestinal symptoms. The assurance of safety, from ongoing search, of cannabinoid-based therapeutic strategies devoid of psychotropic effects can be complemented with local delivery into bladder by intravesical route. A greater understanding of the role of the peripheral CB_1_ and CB_2_ receptor system in lower urinary tract is necessary to allow the development of new treatments.
